# Supplementary material for: Alcohol policies in India: A scoping review
Source: PLoS One. 2023 Nov 17;18(11):e0294392. doi: 10.1371/journal.pone.0294392 (PMC10655994; doi:10.1371/journal.pone.0294392)
Supplement: S1 Table — Outlines policy details obtained during the academic search stage of the review. (DOCX) [file pone.0294392.s001.docx]

| Category | Policy Name (if known) | Policy description | Date Implemented | Jurisdiction/Department Responsible | Citation |
| --- | --- | --- | --- | --- | --- |
| Leadership |  | Alcohol problem prevention efforts |  | Ministry of Social Welfare | (1) |
|  | National Mental Health Programme | District mental health care, upgrade of mental hospitals, increasing specialist human resources; Includes Guiding principles for the treatment of common mental health disorders, which include substance use disorders | 1982 | Federal | (2) |
|  | District Mental Health Programme | Goal of providing community based mental health coverage through decentralized healthcare and reduce inpatient care, expected to cover 36% of districts as of 2015, but varies across districts because of lack of funding absorption and low motivation |  |  | (3) |
|  | Narcotic Drugs and Psychotropic Substances (NDPS) Act, 1985; section 71 | Provides empowerment of the government to establish centres for identification, treatment, education, after care, rehab, social reintegration of substance users, and for supply of any narcotic drugs and psychotropic substance (as prescribed by concerned government) to the users registered with government, and to others where such supply is a medical necessity | 1985 | Federal | (4-6) |
|  | Protection of Women from Domestic Violence Act | "Any act, conduct, omission and comission that harms or injures or has a potential to harm or injure will be considered as Domestic Violence by the law" | 2005 | Federal | (7) |
|  | Central Prohibition Committee, Working Group on Alcohol and Drug Dependence | Group responsible for formulating long-term research activities related to alcohol and drug use in India |  | Federal | (8) |
|  | Persons with Disability Act, 1996 | It appears "alcohol and drug related disability" is included in the act, but not specifically discussed how | 1995 | Federal | (9) |
|  | National Programme for Prevention and Control of Diabetes, Cardiovascular Diseases, and Stroke | One of the objectives of the program is to assess CVD and its risk factors, of which alcohol is one | 2009 | Federal | (10) |
|  | Mental Health Care Act | Use media publicity to reduce the stigma of mental illness and encourage treatment seeking |  | Federal | (11) |
|  | The Protection of Children from Sexual Offences Act | Acts protecting children from several types of abuse | 2012 | Federal | (12) |
|  | National Master Plan | Unclear |  | Federal | (13) |
|  | Mental Health Care Bill, 2013 |  | Not specified - bill was under review at the time of publication |  | (3) |
|  |  | Mass media campaigns to educate about effects of drinking; Welfare Ministry also produced two short films on prohibition that were shown throughout the nation |  | Federal | (14) |
|  |  | Grant program for ngos: 1986-1987 Rs 1.19 given to 24 voluntary orgs for promoting prohibition; |  | Federal | (14) |
| Health Services |  | Treatment for alcohol use disorders offered by governmental organizations |  | Federal | (15) |
|  |  | Treatment of alcohol dependence and sequelae is the responsibility of the Ministry of Health |  | Ministry of Health | (1) |
|  |  | 600 doctors trained to treat alcohol abuse 1999-2009, goal to train another 1000 by 2013, deploy to India's 560 district hospitals |  |  | (16) |
|  | National Drug Deaddiction Programme | Funding for 483 detox and 90 counselling centres across India | 1987-1988  Modified 1992-1993 | Ministry of Health and Family Welfare | (17) |
|  | National Trauma Care Programme | Strengthen trauma care in hospitals and increase number of ambulances on highways to decrease CFR for drink driving related accidents | 11th plan | Federal | (2) |
|  |  | District hospitals provide mental health outreach to primary centers at rural level |  | Federal | (18) |
|  | Scheme for Prohibition and Drug Abuse Prevention | Provision of financial assistance to organisations involved in awareness and preventative education, establishing drug awareness and counselling centres, treatment and rehabilitation centres, and workplace prevention programs and de-addiction camps | Implemented since 1985-1986 | Ministry of Social Justice and Empowerment (MSJandE) | Social cost of alcohol? |
|  | Rashtriya Swasthya Bima Yojana (RSBY) | Health insurance scheme for below poverty line Indians | 2007 |  | (10) |
|  |  | Health-care delivery generally controlled by the states, no central SUD programs supported by national government, and lack of earmarked funding from state governments for SUD treatment |  | Federal and state | (13) |
|  | National Rural Health Mission (NRHM), Indian Public Health Standard (IPHS); Psychiatry Services | IPHS guidelines mention de-addiction centres at the district level, only as a desireable but not an essential service with no standards outlined; as well in the SDH level as an outpatient "follow-up" service; minimum standards set in Psychiatry services section | 2005, IPHS updated in 2010 | Federal | (5) |
|  |  | 400 Integrated Rehabilitation Centres for Addicts (ircas) run by ngos throughout the country |  | Ministry of Social Justice and Empowerment | (5) |
|  | Punjab SUD Treatment and Counseling and Rehabilitation Centres Rules, 2011 (Clause 14) | State specific legislation on detox centres | 2011 | State | (5) |
|  | District Mental Health Programme | Goal of providing community based mental health coverage through decentralized healthcare and reduce inpatient care, expected to cover 36% of districts as of 2015, but varies across districts bc of lack of funding absorption and low motivation | 1996 | Federal | (3) |
| Community |  | Organization of temperance boards |  | States | (15) |
|  |  | Employers pay tribal workers in alcohol (appears to be legal) |  | States | (19) |
|  |  | Ban on liquor sales in tribal areas after local demands |  | States | (20) |
|  |  | Guidelines/policy for the sale and consumption of liquor in tribal areas |  | Federal | (20) |
|  |  | Demands for local control over licensing etc of alcohol in village communities | Unclear if implemented | States | (21) |
| Drink Driving | Motor Vehicle Act | Law prohibiting the use of alcohol use during driving | 1988 | Federal | (22) |
|  | Indian Motor Vehicles Act | Establishment of 0.03g/dl BAC limit |  | State governments (authority granted by Federal government) | (17, 23) |
|  |  | Legalization of breath testing at random road checkpoints |  | Federal | (24) |
|  | Draft national road policy |  | Undergoing approval at time of review | Federal | (2) |
|  | National Urban Transport Policy | Would help slow speeds and reduce accidents including those related to drunk driving |  | Federal | (2) |
| Availability |  | Restriction on the production of 'artisinal illegal spirits' |  | Not specified | (25) |
|  |  | Restriction on the production industrially produced spirits |  | Not specified | (25) |
|  |  | Post-independence alcohol licenses for manufacture and sale were given as political patronage to local party leaders |  | State | (26) |
|  |  | Minimum drinking age |  | State | (27) |
|  |  | Alcohol production licensing |  | State | (28) |
|  |  | Establishment of dry days |  | State | (1) |
|  |  | Restriction of alcohol retailer locations |  | State | (1) |
|  |  | Restriction of alcohol hours of sale |  | State | (1, 29) |
|  |  | Shops/bars/restaurants obtain licenses to sell alcoholic beverages | 1980s-1990s |  | (15) |
|  |  | Alcohol prohibition in whole or part |  | Gujurat, Bihar, Nagaland, Kerala, Tamil Nadu, Manipur | (30-33) |
|  |  | Public drinking restrictions |  | State | (1) |
|  |  | Relaxation of rules of entry for foreign alcohol producers in Indian market including importation and local production under joint ventures | 1990s | Federal | (34) |
|  |  | Licensing of country liquor production |  | State | (35) |
|  | Indian Constitution Article 47 | Directive principle encouraging nationwide alcohol prohibition | 1947 | Federal | (8) |
|  |  | Government controlled alcohol retailers - Kerala State Beverages Corporation |  | State | (19) |
|  |  | Bihar bans alcohol in effort to address problem of domestic violence | 2016 | State | (36) |
|  |  | Ban on sale of alcohol at all highway-side restaurants |  |  | (37) |
|  |  | Prohibition on arrack | 1995 | Andhra Pradesh | (38) |
|  | Andhra Pradesh Excise Act | Following prohibition, the Act was amended to make the manufacture of liquor illegal and punishable with conviction and fines up to Rs100,000 |  | AP | (39) |
|  |  | Establishment of government as the sole distributor of alcohol |  | Tamil Nadu | (35) |
|  | Bombay Prohibition (Gujarat amendment) Act | Amendment to the Bombay Prohibition Act, 1949. The new Act recommends capital punishment or life imprisonment for anyone who participates in manufactoring, selling, or supplying the raw materials for illegal alcohol (latthaand). If the liquour has caused death, imprisionment ranges from 7-10 years. The Act also recommends a Rs.3000 fine and one year imprisionment for police officers who fail to send seized alcohol for testing. |  | Gujurat | (35) |
|  |  | In some states renewal of retail licenses are contingent upon meeting stiff sales quotas |  | State | (17) |
|  | Children Acts omnibus provisions | Adults can be punished for providing alcohol to juveniles and children can come to the attention of the courts for alcohol possession or use |  | Not specified | (40) |
|  |  | Arrests for violations of liquor (prohibition) laws are recorded under the category of "local and special laws" |  | Federal | (40) |
|  |  | Government compensates states for loss of tax revenue from introducing prohibition |  | Federal | (14) |
|  |  | Initiatives to promote wine tourism |  | Ministry of Tourism | (41) |
|  |  | Various forms of government assistance to wine producers including, planting of vines, capital investments, and marketing campaigns |  | State governments | (41) |
| Marketing |  | Alcohol advertising banned in print media |  | Federal | (1) |
|  |  | Alcohol advertising banned in electronic media |  | Federal | (1) |
|  | Cable Television Network Rules | Alcohol advertising banned on cable TV, indirect advertising ("surrogate") still allowed | 1994 | Federal | (42, 43) |
|  |  | Alcohol advertising banned on radio |  | Federal | (44) |
|  |  | Alcohol advertising banned on billboards, lack of enforcement |  | Federal | (44, 45) |
|  |  | Alcohol advertising banned in cinema |  | Federal | (44) |
|  |  | Alcohol advertising banned at point of sale |  | Federal (possibly also state) | (44) |
| Pricing |  | Taxes on alcohol production |  | States | (20) |
|  |  | Taxes on alcohol consumption/sales |  | States | (20) |
|  |  | State tax rates for inputs of country liquor input its prices |  | States | (1) |
|  |  | Minimum alcohol prices set by states |  | States | (12) |
| Negative Consequences |  | Alcohol containers required to carry public health warning |  | Federal | (1) |
|  |  | Government controls licensing, production and alcohol content, IMFL limited to 40% ABV and country liquor 42% ABV |  | Federal | (35) |
| Illicit Alcohol |  | Taxation of illicit alcohol not enforced |  | States | (25) |
|  |  | Laws against production of some locally manufactured alcohol |  | Not specified | (25) |
|  |  | Some state governments offer monetary compensation for death after consumption of poorly made illicit liquor, but as per their discretion |  | States | (35) |
|  | Bombay Prohibition Act | Deals with activities involving manufacting, distilling, selling, buying or transporting lattha | 1949 | Unclear | (35) |
|  | Bombay Prohibition (Gujurat Amendment) Act | Recommends capital punishment for being involved with lattha manufacture/sale | 2009 | Gujurat | (35) |
|  | Bombay Abkari Act, 1878 | Taxation of toddy production and prohibition of other locally produced drinks | 1878 | Federal | (35) |
|  | Mhowra Act, 1892 | Taxation of toddy production and prohibition of other locally produced drinks | 1892 | Federal | (35) |
| Monitoring |  | Ministry of Road Transportation and Highways (MORTH) compiles police data and data on registered vehicles and infrastructure and infrequently publishes it [drink driving] |  | MORTH | (46) |
|  |  | National Crime Records Bureau (NCRB) is most commonly available data in public domain, which is maintained by policy sources at national level [crimes while intoxicated] |  | NCRB | (46) |

1. Saxena S. Alcohol problems and responses: Challenges for India. Journal of Substance Use. 2000;5(1):62-70.

2. Patel V, Chatterji S, Chisholm D, Ebrahim S, Gopalakrishna G, Mathers C, et al. Chronic diseases and injuries in India. Lancet. 2011;377(9763):413-28.

3. Patel V, Xiao S, Chen H, Hanna F, Jotheeswaran A, Luo D, et al. The magnitude of and health system responses to the mental health treatment gap in adults in India and China. The Lancet. 2016;388(10063):3074-84.

4. Saddichha S, Manjunatha N, Khess CRJ. Legislative control of alcohol use: Is it an unmet need of public health policy? Recommendations for India and other developing nations. Journal of Substance Use. 2011;16(5):367-71.

5. Basu D, Avasthi A. Strategy for the management of substance use disorders in the State of Punjab: Developing a structural model of state-level de-addiction services in the health sector (the "punjab model"). Indian Journal of Psychiatry. 2015;57(1):9-20.

6. Benegal V, Velayudhan A, Jain S. The social cost of alcoholism (Karnataka). NIMHANS Journal. 2000;18(1-2):67-76.

7. Aparajita D, Preeti PS, Sahoo SK, Dhiraj B, Amitava K, Madhureema D. Domestic violence and its determinants: a cross-sectional study among women in a slum of Kolkata. Indian Journal of Community Health. 2015;27(3):334-40.

8. Mohan D, Sharma H. International review series: Alcohol and alcohol problems research: VI. India. British Journal of Addiction. 1985;80(4):351-5.

9. Pal HR, Saxena S, Chandrashekhar K, Sudha SJ, Murthy RS, Thara R, et al. Issues related to disability in India: a focus group study. National Medical Journal of India. 2000;13(5):237-41.

10. Arti S, Shikha D. Lower socio-economic status and cardiovascular disease: role of healthcare facility and policy in India. Indian Journal of Community Health. 2016;28(3):215-21.

11. Padhy SK, Khatana S, Sarkar S. Media and mental illness: relevance to India. Journal of Postgraduate Medicine. 2014;60(2):163-70.

12. Esser MB, Rao GN, Gururaj G, Murthy P, Jayarajan D, Sethu L, et al. Physical abuse, psychological abuse and neglect: Evidence of alcohol-related harm to children in five states of India. Drug and Alcohol Review. 2016;35(5):530-8.

13. Ray R, Dhawan A, Chopra A. Addiction research centres and the nurturing of creativity: National Drug Dependence Treatment Centre, India--a profile. Addiction. 2013;108(10):1705-10.

14. Kumar RP. The need for a drug abuse documentation center in India. Bulletin of the Medical Library Association. 1990;78(4):353-7.

15. Bennett LA, Campillo C, Chandrashekar C, Gureje O. Alcoholic beverage consumption in India, Mexico, and Nigeria: A cross-cultural comparison. Alcohol Health & Research World. 1998;22(4):243-52.

16. Prasad R. Alcohol use on the rise in India. The Lancet [Internet]. 2009; 373(9657).

17. Benegal V. India: Alcohol and public health. Addiction. 2005;100(8):1051-4.

18. Chakrabarti A, Rai T, Sharma B, Rai B. Culturally prevalent unrecorded alcohol consumption in Sikkim, North East India: Cross-sectional situation assessment. Journal of Substance Use. 2015;20(3):162-7.

19. Mohindra K, Narayana D, Anushreedha S, Haddad S. Alcohol use and its consequences in South India: Views from a marginalised tribal population. Drug and Alcohol Dependence. 2011;117(1):70-3.

20. Bang AT, Bang RA. Community participation in research and action against alcoholism. World Health Forum. 1991;12(1):104-9.

21. Mishra AK. Tribal women against liquor. Economic and Political Weekly. 1999;34(19):1089-91.

22. Kumar S, Bansal YS, Singh D, Medhi B. Alcohol and drug use in injured drivers - An emergency room study in a regional tertiary care centre of north west India. Journal of Clinical and Diagnostic Research. 2015;9(7):1-4.

23. Esser MB, Wadhwaniya S, Gupta S, Tetali S, Gururaj G, Stevens KA, et al. Characteristics associated with alcohol consumption among emergency department patients presenting with road traffic injuries in Hyderabad, India. Injury. 2016;47(1):160-5.

24. Jiloha RC. Prevention, early intervention, and harm reduction of substance use in adolescents. Indian Journal of Psychiatry. 2017;59(1):111-8.

25. Rehm J, Kailasapillai S, Larsen E, Rehm MX, Samokhvalov AV, Shield KD, et al. A systematic review of the epidemiology of unrecorded alcohol consumption and the chemical composition of unrecorded alcohol. Addiction. 2014;109(6):880-93.

26. Bang A, Bang R. India: Action against sale of alcohol. Lancet. 1992;340(8821):720.

27. Aravind P, Nayak MB, Greenfield TK, Bond JC, Hasin DS, Vikram P. Adolescent drinking onset and its adult consequences among men: a population based study from India. Journal of Epidemiology & Community Health. 2014;68(10):922-7.

28. Das SK, Balakrishnan V, Vasudevan DM. Alcohol: its health and social impact in India. National Medical Journal of India. 2006;19(2):94-9.

29. Priyadarsini S. Public perceptions of drinking by juveniles in India. A case study. Journal of Studies on Alcohol. 1981;42(7):594-603.

30. Kumar S. Price elasticity of alcohol demand in India. Alcohol and Alcoholism. 2017;52(3):390-5.

31. Ningombam S, Hutin Y, Murhekar MV. Prevalence and pattern of substance use among the higher secondary school students of Imphal, Manipur, India. National Medical Journal of India. 2011;24(1):11-5.

32. Varun P. State-sponsored alcoholism in Kerala. Economic and Political Weekly. 2015;50(23):17-20.

33. Subramanian SV, Nandy S, Irving M, Gordon D, Davey Smith G. Role of socioeconomic markers and state prohibition policy in predicting alcohol consumption among men and women in India: a multilevel statistical analysis. Bulletin of the World Health Organization. 2005;83(11):829-36.

34. Skandhan KP. Prohibition of alcohol in India. Lancet. 1992;340(8832):1414-5.

35. Bodwal J, Chauhan M, Ghosh M, Behera C. Hooch tragedies in India: A review. Anil Aggrawal's Internet Journal of Forensic Medicine and Toxicology. 2014;15(1).

36. Sanjeev K, Nishith P. Bihar's alcohol ban: good intentions, impractical policy. Economic and Political Weekly. 2016;51(1):13-5.

37. Sharma DC. India needs shift in thinking to improve road safety. The Lancet. 2015;385(9975):1281-2.

38. Sharma H, Tripathi B, Pelto PJ. The evolution of alcohol use in India. AIDS and Behavior. 2010;14(Suppl 1):S8-S17.

39. Pande R. From anti-arrack to total prohibition: the women's movement in Andhra Pradesh, India. Gender, Technology & Development. 2000;4(1):131-44.

40. Priyadarsini S, Hartjen CA. Legal control and alcohol in the United States and India. International Journal of the Addictions. 1982;17(7):1099-106.

41. Aditi R, Jayashree B. Wine tourism in Maharashtra: problems and solutions. Asian Journal of Food and Agro Industry. 2012;5(2):141-55.

42. Bhaumik S. Campaign group claims that some cricketers in the Indian Premier League are breaching the rules on alcohol advertising. BMJ. 2013;346:f3303.

43. Bonu S, Rani M, Peters DH, Jha P, Nguyen SN. Does use of tobacco or alcohol contribute to impoverishment from hospitalization costs in India? Health Policy & Planning. 2005;20(1):41-9.

44. Moital M, Whitfield J, Jackson C, Bahl A. Event sponsorship by alcoholic and non-alcoholic drinks businesses in India. International Journal of Contemporary Hospitality Management. 2012;24(2):289-311.

45. Jacob KS. Alcohol and public health policies in India. National Medical Journal of India. 2010;23(4):224-5.

46. Barffour M, Gupta S, Gururaj G, Hyder AA. Evidence-based road safety practice in India: assessment of the adequacy of publicly available data in meeting requirements for comprehensive road safety data systems. Traffic Injury Prevention. 2012;13 Suppl 1:17-23.
