# Supplementary material for: Alcohol policies in India: A scoping review
Source: PLoS One. 2023 Nov 17;18(11):e0294392. doi: 10.1371/journal.pone.0294392 (PMC10655994; doi:10.1371/journal.pone.0294392)
Supplement: S2 Table — Outlines the details of the policy documents obtained through all stages of the search at both Federal and State/Territory levels. (DOCX) [file pone.0294392.s002.docx]

| **WHO Global Action Plan Category** | **Implementation Level** | **Policy Name and Citation** | **Policy Section (if applicable** | **Description** |
| --- | --- | --- | --- | --- |
| Leadership | National | Mental Healthcare Act, 2017(1) | N/A | Provides rights to equality and non-discrimination for people with mental illness to the same manner as provided to those with physical illness as well as information on their care and confidentiality of their treatment. Central and State Mental Health Authorities created to review the quality of service provision. |
| Leadership, health services | National | Central Sector Scheme of Assistance for Prevention of Alcoholism and Substance (Drugs) Abuse and For Social Defense Services (2) | N/A | A range of addiction related programs eg education, counselling, de-addiction and rehabilitation as well as grants for NGOs working with those affected by addiction. |
| Leadership, health services | National | Ayushman Bharat | Training Manual on Mental, Neurological and Substance Use (MNS) Disorders Care for Community Health Officer at Ayushman Bharat - Health and Wellness Centres (3) | Guidance for addressing mental health and substance use disorders in the Health and Wellness Centers implemented by the Ayushman Bharat universal healthcare scheme. |
| Health services | National | Juvenile Justice (Care and Protection of Children) Act, 2015 (4) | 93 | Transfer of child from Children's Home who is addicted to alcohol to psychiatric hospital in accordance with Mental Health Act 1987 |
|  |  | Drug Deaddiction Program (5) and “Strengthening the Drug Deaddiction Program: Establishment of DTCs(6) | N/A | Mandates demand reduction for alcohol and drug use; one time grants to establish 122 de-addiction centres (DACs) associated with district hospitals and psychiatry departments establishment of AIIMS as the coordinating body for the DACs; this is strengthened with additional establishment of Drug Treatment Clinics |
|  |  | Indian Public Health Standards, Guidelines for District Hospitals(7) | Psychiatry: alcohol and drug abuse | Specifies guidelines of treatment and follow-up, and if possible management |
|  | Subnational | Punjab Substance Use Disorder Treatment and Counseling and Rehabilitation Centres Rules, 2011 (8) |  | Sets specific standards for substance use disorder treatment in Punjab |
| Community | National | The Provisions Of The Panchayats (Extension To The Scheduled Areas) Act, 1996 (9) | m(i) | Specifically endows the Scheduled Areas panchayats the power to enforce prohibition or to regulate or restrict the sale and consumption of any intoxicant |
| Drink Driving | National | Motor Vehicles Act 1988 (10) | 185 | BAC limit 30 mg per 100 ml test by breath analyser with punishments |
|  |  | Motor Vehicles Act 1988 (10) | 203 | Uniformed officers can breathylyze anyone they think reasonably intoxicated including after accidents, in hospitals |
|  | National | Ban on liquor shops along National Highways (11) |  |  |
| Availability | National | Juvenile Justice (Care and Protection of Children) Act, 2015 (4)  Amendment Act, 2021(12) | 77 | Penalty for giving liquor to child |
|  |  | Juvenile Justice (Care and Protection of Children) Act, 2015 (4)  Amendment Act, 2021(12) | 78 | Penalty for using child for peddling liquor |
|  |  | The Cantonments Act(13) | 285 | No unauthorized sale of liquor within cantonments, details fine and imprisonment |
|  |  | The Cantonments Act(13) | 286 | no possession of liquor except by military officers within cantonments; details fine and imprisonment |
|  | Subnational | The Delhi Police Act 1978(14) | 112 | Penalty for not obtaining a licence to operate a place of public entertainment (any kind of place which serves liquor) |
| Marketing | National | Cable Television Networks (Amendment) Rules, 2021 (15) | 2 | Cable advertisements cannot refer to alcohol products, but brands can still be used |
|  |  | Norms of Journalistic Conduct, 2020 (16) | 36(ii) | No advertisements of liquor on written media |
|  |  | Code for Commercial Advertising, All India Radio (17) | 2(vi) | No advertisements of liquor on radio |
| Pricing | National | The Goods and Services Tax (Compensation to States) Act (18) | 5 (a)(b)(c) | state refunds for GST did not include any tax levied on alcohol in its calculation of base revenue |
|  |  | The Integrated Goods and Services Tax Act, 2017 (19) | 5 | liquor excluded from GST |
|  |  | The Union Territory Goods and Services Tax, 2017 (20) | 7 | liquor excluded from GST |
|  |  | The Income Tax Act (21) | 206C | One percent income tax on profits/gains from selling alcohol |
|  | Subnational | The Delhi Sales Tax Act 1975 (22) | 4(1)(a) | twelve paise turnover tax on liquor (country-made and IMFL) in UT of Delhi |
| Neg consequences | National | Food Safety and Standards (Alcoholic Beverages) Regulations, 2018 (23) | N/A | labelling requirements for contents and standards requirements for ingredients of alcoholic beverages |
|  |  | Protection of Women from Domestic Violence Act (24) | N/A | appoints the duties and rights of various stakeholders in the event of domestic violence (associated with alcohol intoxication) |
|  |  | Capacity Building for developing Trauma Facilities on National Highways (25) | N/A | Upgrading of 85 healthcare facilities and finishing pending facilities to set up a robust trauma registry and injury surveillance system with a goal to decrease preventable deaths from road accidents |
|  |  | National Urban Transport Policy, 2014 (26) | N/A | better urban planning and decreasing motor vehicle congestion, one goal to decrease motor vehicle accidents |

Excise Acts

| **WHO Global Action Plan Category** | **Policy** | **States/Territories Where These Policies Are Present in Most Recent Excise Act** |
| --- | --- | --- |
| Availability | Prohibition | Bihar, Gujarat, Nagaland, Mizoram, Manipur |
|  | Import regulation | Andhra Pradesh, Arunachal Pradesh, Assam, Bihar, Daman & Diu, Goa, Gujarat, Haryana, Himachal Pradesh, Odisha, Nagaland, Madhya Pradesh, Maharashtra, Jammu and Kashmir, Jharkhand, Karnataka, Andaman and Nicobar Islands, Dadra and Nagar Haveli, Punjab, Chandigarh, Rajasthan, Tamil Nadu, Tripura, Uttar Pradesh, West Bengal, Mizoram, Sikkim, Puducherry, Telangana, Manipur, Delhi, Kerala, Uttarakhand |
|  | Export Regulation | Andhra Pradesh, Arunachal Pradesh, Assam, Bihar, Daman & Diu, Goa, Gujarat, Haryana, Himachal Pradesh, Odisha, Nagaland, Madhya Pradesh, Maharashtra, Jammu and Kashmir, Jharkhand, Karnataka, Andaman and Nicobar Islands, Dadra and Nagar Haveli, Punjab, Chandigarh, Rajasthan, Tamil Nadu, Tripura, Uttar Pradesh, West Bengal, Mizoram, Sikkim, Puducherry, Telangana, Manipur, Delhi, Kerala |
|  | Transport regulation | Andhra Pradesh, Arunachal Pradesh, Assam, Bihar, Daman & Diu, Goa, Gujarat, Haryana, Himachal Pradesh, Odisha, Nagaland, Madhya Pradesh, Maharashtra, Jammu and Kashmir, Jharkhand, Karnataka, Andaman and Nicobar Islands, Dadra and Nagar Haveli, Punjab, Chandigarh, Rajasthan, Tamil Nadu, Tripura, Uttar Pradesh, West Bengal, Mizoram, Sikkim, Puducherry, Telangana, Manipur, Delhi, Kerala, Uttarakhand |
|  | Licensing of manufacture | Andhra Pradesh, Arunachal Pradesh, Assam, Bihar, Daman & Diu, Goa, Gujarat, Haryana, Himachal Pradesh, Odisha, Nagaland, Madhya Pradesh, Maharashtra, Jammu and Kashmir, Jharkhand, Karnataka, Andaman and Nicobar Islands, Dadra and Nagar Haveli, Punjab, Chandigarh, Rajasthan, Tamil Nadu, Tripura, Uttar Pradesh, West Bengal, Mizoram, Sikkim, Puducherry, Telangana, Manipur, Delhi, Kerala, Uttarakhand |
|  | Ban on sales/drinking in public spaces | Assam, Bihar, Daman & Diu, Goa, Gujarat, Haryana, Himachal Pradesh, Nagaland, Maharashtra, Jharkhand, Karnataka, Andaman and Nicobar Islands, Punjab, Chandigarh, Rajasthan, Manipur, Delhi, Kerala |
|  | Licensing of places of sale/consumption | Andhra Pradesh, Arunachal Pradesh, Assam, Bihar, Daman & Diu, Goa, Gujarat, Haryana, Himachal Pradesh, Odisha, Nagaland, Madhya Pradesh, Maharashtra, Jammu and Kashmir, Jharkhand, Karnataka, Andaman and Nicobar Islands, Dadra and Nagar Haveli, Punjab, Chandigarh, Rajasthan, Tamil Nadu, Tripura, Uttar Pradesh, West Bengal, Mizoram, Sikkim, Puducherry, Telangana, Manipur, Delhi, Kerala, Uttarakhand |
|  | Regulation of days or hours of sale | Andhra Pradesh, Arunachal Pradesh, Assam, Daman & Diu, Goa, Haryana, Odisha, Nagaland, Madhya Pradesh, Maharashtra, Jammu and Kashmir, Jharkhand, Andaman and Nicobar Islands, Dadra and Nagar Haveli, Punjab, Chandigarh, Rajasthan, Tamil Nadu, Tripura, West Bengal, Sikkim, Puducherry, Telangana, Delhi, Kerala, Uttarakhand |
|  | Quota for retail sale or personal possession limit | Andhra Pradesh, Arunachal Pradesh, Assam, Daman & Diu, Goa, Gujarat, Haryana, Himachal Pradesh, Madhya Pradesh, Maharashtra, Jammu and Kashmir, Jharkhand, Karnataka, Punjab, Chandigarh, Rajasthan, Tripura, Uttar Pradesh, West Bengal, Puducherry, Telangana, Delhi, Kerala, Uttarakhand |
|  | Outlet density regulations | Arunachal Pradesh, Haryana, Himachal Pradesh, Odisha, Madhya Pradesh, Maharashtra, Jammu and Kashmir, Karnataka, Dadra and Nagar Haveli, Rajasthan, Tamil Nadu, Tripura, Puducherry, Telangana |
|  | Place of sale regulation | Arunachal Pradesh, Assam, Bihar, Daman & Diu, Goa, Gujarat, Haryana, Himachal Pradesh, Odisha, Nagaland, Madhya Pradesh, Maharashtra, Jharkhand, Karnataka, Andaman and Nicobar Islands, Dadra and Nagar Haveli, Punjab, Chandigarh, Tamil Nadu, Tripura, Uttar Pradesh, West Bengal, Sikkim, Puducherry, Telangana, Uttarakhand |
|  | State distribution system for alcoholic beverages | Andhra Pradesh, Gujarat, Tamil Nadu, Telangana, Kerala, Jharkhand |
|  | Minimum age | 18 years: Assam, Bihar, Himachal Pradesh, Odisha, Karnataka, Andaman and Nicobar Islands, Rajasthan, Uttar Pradesh, Sikkim, Puducherry, Kerala  21 years: Andhra Pradesh, Arunachal Pradesh, Daman & Diu, Goa, Madhya Pradesh, Haryana, Jammu and Kashmir, Jharkhand, Dadra and Nagar Haveli, Tamil Nadu, Tripura, West Bengal, Telangana, Uttarakhand  25 years: Punjab, Delhi  26 years: Chandigarh  No sale to “minors”: Maharashtra |
| Marketing | State regulations or ban on advertisement, promotion and sponsorship | Assam, Bihar, Daman & Diu, Goa, Gujarat, Haryana, Himachal Pradesh, Nagaland, Madhya Pradesh, Maharashtra, Jammu and Kashmir, Andaman and Nicobar Islands, Dadra and Nagar Haveli, Punjab, Chandigarh, Rajasthan, Tamil Nadu, Uttar Pradesh, Mizoram, Manipur, Delhi |
|  | Point of sale advertising ban | Daman & Diu, Goa, Gujarat, Haryana, Himachal Pradesh, Nagaland, Madhya Pradesh, Maharashtra, Jammu and Kashmir, Andaman and Nicobar Islands, Dadra and Nagar Haveli, Punjab, Chandigarh, Rajasthan, Tamil Nadu, Tripura, Uttar Pradesh, Mizoram, Manipur, Delhi |
| Negative consequences | Warnings or security hologram | Bihar, Haryana, Odisha, Jammu and Kashmir, Jharkhand, Chandigarh, Tamil Nadu |
| Illicit and Informal Alcohol | Penalties for violation of act (for example unlawful manufacture, import, or sale of alcohol) | Andhra Pradesh, Arunachal Pradesh, Assam, Daman & Diu, Goa, Gujarat, Uttar Pradesh, West Bengal, Mizoram, Puducherry, Orissa, Nagaland, Madhya Pradesh, Meghalaya, Maharashtra, Jharkhand, Karnataka, Andaman and Nicobar Islands, Dadra and Nagar Haveli, Punjab, Rajasthan, Tamil Nadu |
|  | Informal alcohol production/consumption permitted with regulation | Andhra Pradesh, Assam, Daman & Diu, Goa, Himachal Pradesh, Telangana, Uttar Pradesh, Orissa, Madhya Pradesh, Meghalaya, Maharashtra, Jharkhand, Karnataka, Andaman and Nicobar Islands, Dadra and Nagar Haveli, Punjab, Rajasthan, |
|  | Informal alcohol production banned | West Bengal, Mizoram, Kerala, Tamil Nadu, Nagaland |
| Pricing | Minimum/maximum sale price | Haryana, Punjab, Chandigarh, Rajasthan, West Bengal, Sikkim |

**General Policy References**

1. The Mental Healthcare Act, (2017).

2. Central Sector Scheme of Assistance for Prevention of Alcoholism and Substance (Drugs) Abuse and For Social Defense Services, (2015).

3. Training Manual on Mental, Neurological and Substance Use (MNS) Disorders Care for Community Health Officer at Ayushman Bharat - Health and Wellness Centres. In: Ministry of Health and Family Welfare, editor. 2021.

4. The Juvenile Justice (Care and Protection of Children) Bill, (2015).

5. Ministry of Health and Family Welfare. Drug De-addiction Programme (DDAP).

6. National Drug Dependence Treatment Centre A. Strategy and Action Plan: Enhancing the functioning of Drug Deaddiction Centers under DDAP. In: Ministry of Health and Family Welfare, editor. New Delhi, India.

7. Indian Public Health Standards: Guidelines for District Hospitals. In: Directorate General of Health Services MoHFW, editor. New Delhi, India2012.

8. The Punjab Sunstance Use Treatment & Counselling and Rehabilitation Centers Rules, (2011).

9. The Provisions of the Panchayats (Extension to Scheduled Areas) Act, (1996).

10. The Motor Vehicles Act, (1988).

11. Ban on liquor shops along National Highways., (2017).

12. The Juvenile Justice (Care and Protection of Children) Amendment Act, 2021, (2021).

13. The Cantonments Act, (2006).

14. The Delhi Police Act, (1978).

15. Cable Television Networks (Amendment) Rules, 2021.

16. Norms of Journalistic Conduct, (2020).

17. Code for Commercial Advertising.

18. The Goods and Services Tax (Compensation to States) Act, (2017).

19. The Integrated Goods and Services Tax Act, (2017).

20. The Union Territory Goods and Services Tax, 2017, (2017).

21. Income Tax Act, (1961).

22. The Delhi Sales Tax Act, (1975).

23. Food Safety and Standards (Alcoholic Beverages) Regulations, (2018).

24. Protection of Women from Domestic Violence Act, (2005).

25. Capacity Building for developing Trauma Facilities on National Highways. In: Welfare MoHF, editor. New Delhi, India2014.

26. National Urban Transport Policy. In: Transport MoU, editor. New Delhi, India2014.

**Excise Acts References**

1. The Bengal Excise Act. Excise Department; 1909.

2. The United Provinces Excise Act. Department of Excise; 1910.

3. Eastern Bengal and Assam Excise Act. Directorate of Excise; 1910.

4. The Punjab Excise Act. Department of Excise and Taxation; 1914.

5. The Excise Law of Bihar and Jharkhand. Department of Excise and Prohibition; 1915.

6. The MP Excise Act. Department of Commercial Taxes; 1915.

7. The Tamil Nadu Prohibition Act. Department of Home, Prohibition and Excise; 1937.

8. The Gujurat Prohibition Act. Department of Home; 1949.

9. The Rajasthan Excise Act. Department of Excise; 1950.

10. The Jammu and Kashmir Excise Act. Department of Finance; 1958.

11. The Goa Excise Duty Act. Department of Excise; 1964.

12. The Karnataka Excise Act. Department of Finance; 1965.

13. The Abkari Act, 1 of 1077. Abkari Department; 1967.

14. The Telangana Excise Act. Department of Revenue; 1968.

15. Andhra Pradesh Excise Act. 1968.

16. The Puducherry Excise Act. Department of Law; 1970.

17. The Meghalaya Intoxicating Liquor (Prohibition of Publication of Advertisement) Act. Department of Excise; 1976.

18. The Tamil Nadu Disposal of Articles (Confiscated Under the Tamil Nadu Prohibition Act) Rules. Department of Home, Prohibition and Excise; 1979.

19. The Tamil Nadu Indian Made Foreign Spirits (Manufacture) Rules. Department of Home, Prohibtion and Excise; 1981.

20. Tamil Nadu Liquor (License and Permit) Rules. Department of Home, Prohibition and Excise; 1981.

21. The Tamil Nadu Liquor (Transit) Rules. Department of Home, Prohibition and Excise; 1982.

22. The Tamil Nadu Brewery Rules. Department of Prohibition and Excise; 1983.

23. Tamil Nadu Mass Wine Rules. Department of Home, Prohibition and Excise; 1984.

24. The Tripura Excise Act. Department of Commissioner of Taxes; 1987.

25. The Sikkim Excise Act. Department of Excise (Abkari); 1992.

26. The Arunachal Pradesh Excise Act. 1993.

27. The Nagaland Liquor Total Prohibition (1st Amendment) Act Department of Excise; 1994.

28. The Assam Excise Act. 2000.

29. The Manipur Liquor Prohibition (Amendment) Act. Department of Finance; 2002.

30. The Tamil Nadu Retail Vending (In Shops and Bars) Rules. Department of Home, Prohibition and Excise; 2003.

31. Tamil Nadu Wine (Manufacture) Rules. Department of Prohibition and Excise; 2006.

32. The Delhi Excise Act. Department of Excise, Entertainment and Luxury Tax; 2009.

33. The Himachal Pradesh Excise Act. Ministry of Excise and Taxation; 2011.

34. The Dadra and Nagar Haveli Excise Regulation. Department of Excise; 2012.

35. The Andaman and Nicobar Islands Excise Regulation Department of Finance; 2012.

36. The Odisha Excise Act. Department of Law; 2013.

37. The Jharkhand Excise Amendment Act. Department of Excise and Prohibition; 2015.

38. The Punjab Excise (Amendment) Act. Department of Excise and Taxation; 2017.

39. The Odisha Excise Rules. Department of Excise; 2017.

40. The Excise Policy of U.T Chandigarh for the Year 2018-19. Department of Excise and Taxation; 2018.

41. Jharkhand Excise (Country Liquor Manufacture, Bottling and Storage) Rules. Department of Excise and Prohibition; 2018.

42. Bombay Act No.XXV of 1949, The Maharashtra Prohibition Act. Department of Excise; 2018.

43. Bihar Prohibition and Excise (Amendment) Act. Department of Registration, Excise and Prohibition; 2018.

44. Uttrakhand Excise Policy Subject Matter Manual. Department of Excise Duty; 2019.

45. The Mizoram Liquor (Prohibition) Act. Law and Judicial Department; 2019.

46. Excise Policy, 2019-20. Department of Excise; 2019.

47. Haryana Excise Policy for the Year 2019-20. 2019.

48. Madhya Pradesh Excise (Amendment) Bill. Department of Excise; 2021.
