# Supplementary material for: Alcohol policies in India: A scoping review
Source: PLoS One. 2023 Nov 17;18(11):e0294392. doi: 10.1371/journal.pone.0294392 (PMC10655994; doi:10.1371/journal.pone.0294392)
Supplement: Attachment — Submitted filename: Response to Reviewers.docx [file pone.0294392.s003.docx]

**RESPONSE TO REVIEWERS’ COMMENTS**

**Reviewer 1:**

1. Scoping review methodology should be clearly stated including reporting checklists(guideline)

*We have given a summary of the methodology in the methods section and have cited directly to our published protocol which gives full detail on our methods (can be found* [*here*](https://bmjopen.bmj.com/content/bmjopen/8/12/e020854.full.pdf)*). We chose this approach to conserve word count. However, if Reviewer 1 and the Journal Editor wishes us to also include the full details in this paper we will of course do so. There are no reporting checklists specific to the scoping review methodology, but we have used the PRISMA reporting approach for the academic review stage and this is included in the paper as Figure 2.*

2. Eligibility criteria for the selection of previously existed alcohol policies should be clearly stated.

*The criteria are stated in our protocol, as discussed above, but as with the previous comment, we are happy to include these details here too, as long as the extra words are allowed.*

3. You have to use the updated references, i.e reference 14,24,31 and 32

*We are unclear on what the reviewer means by using updated references.  If this has not otherwise been addressed by responses and revisions to other reviewers’ comments, can we please get some clarity on this comment?*

**Reviewer 2:**

The review is comprehensive and methodology is sound. But the authors are suggested to cite references in the texts of results section in addition to S1 Table. It is also needed to check spelling at the line 174 for surveillance and confirm the phrase "abv or as proof" at line 340.

*References have been added to the results section to cite directly to policy documents mentioned. Spelling has been corrected on line 174 (now line 186) and “abv” has been expanded to “alcohol by volume” on line 340 (now line 370).*

**Reviewer 3:**

A critical policy review. This a reasonable effort from the authors, as covering a large amount of grey literature on the topic, is a tough job.

The major drawback is not repeating the academic search after 2017. In 6 years, as the authors pointed out and also mentioned the impact of COVID-19, there has been research on the impact of the lack of alcohol policy during COVID-19. Authors should consider repeating the academic search. Though you have stated, "because the sole reason for doing that search was to identify policies, and we considered that the updating of the policy search described below would reveal any new or revised policies" But, your objective also includes impact on societal outcomes, how will you know that societal outcomes just from policy documents?

*As described on lines 106-109 our objective was to define* ***the policies which regulate*** *alcohol supply and demand and related societal impacts. We have clarified the language to make this clear. We are* ***not*** *reviewing evidence on the societal impacts of alcohol use. Therefore, as our academic search remains just a step towards identifying relevant policies, we still believe that the updated policy search is sufficient. We agree that if we* ***were*** *undertaking a review of ‘the impact of alcohol policies on societal outcomes’, then we would have needed to repeat – but if we had been undertaking* ***that*** *review (of ‘the impact of alcohol policies on societal outcomes’ then this would have been a very different paper.*

1. Although the authors stated the objective of the impact of societal outcomes, I could only see a little information in the results or discussion in a structured way.

*As stated above, we have clarified the language to make clear that we are interested in policy which* ***regulates*** *the societal impact of alcohol use. This is NOT a review of societal impact, and it was not our intention to have as an objective an examination of the impact of policies ON societal outcomes.  The review is to* ***clarify what policies are in existence****.  These policies are set out as intending to have a societal impact, but this review is not examining the results of the intentions of the policies – it is simply trying to examine what policies are there.*

2. You found significant gaps. How did you find the gaps? What parameter is used? It is unclear to the reader how the authors concluded.

*Our research question called for identifying existing policies, not identifying gaps. Our results highlight the existence of policies across 10 categories as outlined by the WHO Global Strategy to Reduce the Use of Alcohol. We use the discussion to put the identified policies in context, given the recommendations of the Global Strategy.*

3. Where does the country stand on the alcohol policy index based on the author's assessment, is there a change that the authors noted based on the assessment from previous studies? Ref: <https://doi.org/10.1371/journal.pmed.0040151>

*The goal of our paper is not to compare the alcohol policies in India to other countries, so we have not used the Alcohol Policy Index or other scoring type approaches in this paper.*

4. Leadership, awareness and commitment: What about NAPDDR 2018 (national action plan on drug demand reduction 2018), RPWD 2016 including mental illness (also substance use).

*The NAPDDR 2018 is an implementation plan following up on the Narcotic Drug and Psychotropic Substance (NDPS) Act. Alcohol is not included in the definition of psychotropic substances in this act and the NAPDDR does not mention alcohol in the context of drug demand reduction strategies. We thank Reviewer 3 however for drawing this to our attention, and we have added this to our discussion, because it further emphasizes that there is further investigation needed into the* ***implementation*** *of policy, beyond what is included in the text of policy documents on lines 524-534.*

*Regarding the RPWD 2016, substance use is not included as one of 21 disabilities covered in this act and, further, there is no regulation of the use/misuse of alcohol in this act. It is therefore not in scope for this review.*

5. Health services’ response: What about DTC program, ATF, Ayushman bharath health and wellness centres, Nasha mukht Bharath Abayan?

*Again we thank Reviewer 3: we appreciate this comment to make sure our review is thorough. Nasha Mukht Bharath Abayan is an element of the NAPDDR which, as previously discussed, is out of scope of our search. The DTC program is covered by the DDAP program mentioned in the health services result section. We were able to find an operational manual for MNS disorders for the Ayushman Bharat Health and Wellness Centres and have added this to the health services results.*

6. Under community action, NAPDDR 2018 use CLPI community lead peer intervention <https://grants-msje.gov.in/omrunningcpli>

*As discussed above, the NAPDDR is out of scope for our search as it does not specify a policy response to alcohol use.*

7. Drinking and driving policies and counter measures: Ban on alcohol sales 500m on national highways: <https://morth.nic.in/sites/default/files/circulars_document/SR-2017.06.01-Ban%20on%20liquor%20shops%20along%20National%20Highways.pdf>

*Again we thank Reviewer 3: we have added this policy to this section as suggested. This can be found on lines 252-253.*

8. Under illicit alcohol: what about unrecorded alcohol use and related policies? <https://www.iard.org/getattachment/fdd90791-41cb-4bd3-98f0-555fbf9818f8/unrecorded-alcohol-in-india.pdf>

*We fully agree that unrecorded alcohol use is a major issue. Unrecorded alcohol use may be impacted by policies on availability of alcohol, monitoring and surveillance policies, as well as policies to reduce the public health impact of illicit alcohol use. Still, we did not find policy documents which addressed the issue of unrecorded alcohol directly.*

9. Under monitoring and surveillance: Government of India monitors, regularly updates the excise related crime and enforcement process as a part of supply reduction <https://ncrb.gov.in/sites/default/files/CII-2021/CII_2021Volume%201.pdf>

*We have focused on policy documents and their specific contents. We have not found a policy which specifies this as a government directive and have therefore not included this activity in our results. However, we have added this activity to our discussion, to highlight the disparity between written policy and implemented practices, on lines 523-526.*

10. Finally, the overall discussion appears towards the overall (hybrid) system, maybe gaps in comparison to global standards, deviation or less evidence informed policies needs to be depicted. Further, there is a significant amount of grey literature available, authors should be congratulated for their efforts in covering such a vast literature, a final round of consultation with policy experts might improve further.

*Thank you very much for this comment. We believe we have sufficiently covered all areas of concern raised in this point. In our discussion, we first identify that there are gaps which exist in comparison to global standards using the WHO Global Strategy, highlighting where the policy in India is in line with recommendations and where there are still areas to improve evidenced-based approaches. The gap in evidenced-based approaches mostly comes from the diversity across the country, given the hybrid system, and we therefore then investigate the advantages and disadvantages of the system. We have included a round of consultation in our methods and have followed the methods in our protocol to identify policy documents (while acknowledging the limitations of our methods as well in this section) and therefore have not conducted an additional round of consultation as part of this revision.*
